# The cost-effectiveness of standalone HEPA filtration units for the prevention of airborne SARS CoV-2 transmission

**DOI:** 10.1186/s12962-022-00356-1

**Published:** 2022-05-12

**Authors:** Zafar Zafari, Pedro M. de Oliveira, Savvas Gkantonas, Chinenye Ezeh, Peter Alexander Muennig

**Affiliations:** 1 Department of Health Services Research, University of Maryland School of Pharmacy, Baltimore, MD 21201 USA; 2 Department of Engineering, University of Cambridge, Cambridge, UK; 3 Mailman School of Public Health, Columbia University, New York, NY USA

**Keywords:** Economic evaluation, Improving ventilation, Covid-19, SARS-CoV-2, Commercial spaces, Restaurants and bars, Prevention strategies

## Abstract

**Objective:**

Airborne infection from aerosolized SARS-CoV-2 poses an economic challenge for businesses without existing heating, ventilation, and air conditioning (HVAC) systems. The Environmental Protection Agency notes that standalone units may be used in areas without existing HVAC systems, but the cost and effectiveness of standalone units has not been evaluated.

**Study design:**

Cost-effectiveness analysis with Monte Carlo simulation and aerosol transmission modeling.

**Methods:**

We built a probabilistic decision-analytic model in a Monte Carlo simulation that examines aerosol transmission of SARS-CoV-2 in an indoor space. As a base case study, we built a model that simulated a poorly ventilated indoor 1000 square foot restaurant and the range of Covid-19 prevalence of actively infectious cases (best-case: 0.1%, base-case: 2%, and worst-case: 3%) and vaccination rates (best-case: 90%, base-case: 70%, and worst-case: 0%) in New York City. We evaluated the cost-effectiveness of improving ventilation rate to 12 air changes per hour (ACH), the equivalent of hospital-grade filtration systems used in emergency departments. We also provide a customizable online tool that allows the user to change model parameters.

**Results:**

All 3 scenarios resulted in a net cost-savings and infections averted. For the base-case scenario, improving ventilation to 12 ACH was associated with 54 [95% Credible Interval (CrI): 29–86] aerosol infections averted over 1 year, producing an estimated cost savings of $152,701 (95% CrI: $80,663, $249,501) and 1.35 (95% CrI: 0.72, 2.24) quality-adjusted life years (QALYs) gained.

**Conclusions:**

It is cost-effective to improve indoor ventilation in small businesses in older buildings that lack HVAC systems during the pandemic.

## Introduction

SARS-CoV-2 may be transmitted person-to-person via exhaled respiratory aerosols that accumulate within poorly ventilated spaces [1–5]. Airborne transmission of SARS-CoV-2 therefore poses major public health and economic challenges as commercial spaces re-open [3]. To meet this challenge, newer commercial spaces can upgrade existing heating, ventilation, and air conditioning (HVAC) systems [6–9]. One standard is 12 air exchanges per hour (ACH), the recommended ventilation for emergency department waiting rooms [10].

However, older buildings tend not to have HVAC systems installed. When HVAC systems are not present, the Environmental Protection Agency (EPA) recommends alternative means of disinfecting the air [11]. These may include standalone filtration systems containing high efficiency particulate air (HEPA) or electrostatic units, which filter particles down to the sub-micrometer size [11]. However, standalone HEPA filtration units are not designed for high-volume air filtration, and the size and quantity of such units to achieve 12 ACH may not be practical. This is particularly true in poorly ventilated bars, cafes, and restaurants because customers intermingle without face coverings [12, 13].

Given that standalone HEPA filtration units may be only marginally effective and are relatively expensive, we evaluated their cost-effectiveness. In this paper, we provide data for an example setting (a poorly ventilated restaurant), but our online model can be modified for any scenario.

## Methods

We built a decision-analytic model that is designed to assist local and federal regulators in setting standards for improving the indoor air ventilation in poorly ventilated indoor commercial spaces for the prevention of SARS-CoV-2 infections via aerosolized particles. The model is designed to compute the incremental cost-effectiveness ratio (ICER), which is the net cost of an intervention divided by the number of quality-adjusted life years (QALYs) gained [14]. The ICER can be used to compare commonly deployed health or medical interventions to assess whether they are affordable [15, 16]. We followed guidelines for conducting our cost-effectiveness analyses [17], including estimation of costs from a societal perspective. We based our analysis on a standardized space for the purposes of this paper. We based the range of actively infectious cases and full vaccination rates from New York City between March 1, 2020 through March 1, 2022. More importantly, we provided an online interface that allows the user to tailor the model input parameters. This online model can be used to estimate the cost-effectiveness of improving the indoor ventilation rate over a range of costs, spaces, and viral transmission rates (https://openupuniversities.shinyapps.io/Airborne_Transmission_Covid19/).

### Characteristics of the standardized space

Each restaurant, café, or bar is unique with respect to the size, number of customers, hours of operation, and the time that customers spend in the establishment. This variation presents challenges for understanding the airflow and filtration needs for any given business. In this paper, we used a small, poorly ventilated restaurant space as an example so that the reader can get a general idea of the cost-effectiveness of standalone ventilation. In addition, our customizable interface can allow both regulators and restaurant owners to obtain estimates for a range of settings.

The standardized restaurant was open for a total of 3 h for lunch service and 6 h for dinner service. We assumed that the restaurant has a seating capacity of 30 occupants in a 1000 square foot space and a ceiling height of 9 feet, and that each occupant is seated for one-hour at lunch and 1.5-h during dinner. The model assumptions are listed in Table 1.

**Table 1:** Model assumptions for evaluating the cost-effectiveness of improving ventilation in commercial spaces for the prevention of SARS-CoV-2

Assumptions
The standardized room of 1000 square-foot with a ceiling height of 9 feet has 0.8 air changes per hour, primarily from the door opening and closing and the food vent running
For lunch, the restaurant is open for 3 h. Each of 30 occupants is seated for one hour. We modeled 3 consecutive lunch events each for a duration of 1 h. In each event, the restaurant is at the full seating capacity
For dinner, the restaurant is open for 6 h. Each of 30 occupants is seated for 1.5 h. We modeled 4 consecutive dinner events each for a duration of 1.5 h. In each event, the restaurant is at the full seating capacity
Between lunch and dinner hours, the restaurant is closed for enough time so that the virus concentration in the indoor air dropped to zero as workers opened doors and moved throughout the space
The restaurant is operating 7 days a week with similar lunch and dinner hours
The model is built under well-mixed conditions for an infected individual present in an indoor space and there is dynamic airflow in unpredictable patterns associated with the movement of people and an overhead fan [18, 19]
We assumed that transmission through the close-range mode—that is, when infectious aerosols were inhaled directly from the exhaled breath of an infected individual by a susceptible person in its vicinity—is on par between the comparison arms. Thus, only infection through the inhalation of accumulated aerosols, often referred to as the long-range mode of airborne transmission, is modeled and close-range transmission is not modeled [18, 19]
We assumed that infected symptomatic Covid-19 cases would quarantine for 14 days. We also assumed those infected cases who required hospitalizations would quarantine for 21 days
All wages were valued at the median hourly wage in the US [14]

*US* United States

### Temporal evolution of concentration of viable viral copies in an indoor space

In a previous published work, two co-authors developed a model [18] and online tool [19] for the temporal concentration of aerosolized viral copies in the air under well-mixed conditions for an infected individual present in an indoor space. It should be noted that stratification of infectious aerosols and, therefore, a deviation from well-mixed conditions, will occur across different indoor settings. Differences in room geometry, temperature, or positioning of the HEPA units will impact the results [20]. However, the motion of occupants [21] characteristic of busy settings such as those investigated in this work adds mixing to the room air leading to a condition closer to well-mixed aerosols [22]. Specialist advice should be taken when positioning such standalone units. The model considers the evaporation and settling of virus-laden droplets of various sizes exhaled by an infected individual in terms of plaque forming units (PFUs). These are evaluated from a combination of reduced-order modeling and previous experimental measurements. The details are described elsewhere [18]. In brief, the concentration of PFUs dispelled by an infected person in an indoor space can be shown as:

$$C\left( t \right) = n_{\inf } \cdot \frac{{N_{gen} }}{{V \cdot \left( {\lambda + \kappa + \nu + s} \right)}} + \left[ {C\left( {t_{0} } \right) - n_{\inf } \cdot \frac{{N_{gen} }}{{V \cdot \left( {\lambda + \kappa + \nu + s} \right)}}} \right] \cdot \exp \left[ { - \left( {\lambda + \kappa + \nu + s} \right) \cdot \left( {t - t_{0} } \right)} \right],$$

where, $C\left(t\right):$ concentration of viral PFUs over time and $C\left({t}_{0}\right)$ represents the concentration at baseline; ${n}_{inf}:$ number of infected individuals in the room; ${N}_{gen}:$ generating factor for viral particles emitted by continuous exhalation of the infected person while speaking per time unit. The generating factor of 0.059 PFU/second [18] was estimated using a viral load at the sputum of the infected person of 10^10^ virus RNA copies/ml. While the mean virus RNA copies/ml of the infected sputum for the original strains of Covid-19 was estimated as 7 × 10^6^ [23], the emerging evidence shows that the number of viral copies is almost 1000 times larger for the Delta variant [24], the most common Covid-19 strain at the time of publication. Therefore, to be conservative, we assumed a 10^10^ virus RNA copies/ml for the Delta variant. We then applied a conversion factor of 0.01 to estimate PFUs (infectious units) from RNA copies [18]. Here, the aerosol cut-off diameter—the size below which particles are carried by the ventilation air flow—was assumed to be 20 μm. The exhalation flow rate was assumed to be 0.211 l/second representing a sedentary activity [18]; $V:$ Volume of the room. We showed our analysis for a 1000 ft^2^ restaurant area size with a ceiling height of 9 ft; $\lambda :$ Natural viral decay rate. An exponential decay at a rate of 0.636 per hour was assumed [25]; $\kappa :$ Settling rate of aerosols by gravity. A value of 0.39 per hour was assumed [18]; $\nu :$ ventilation rate of the room with outside air; and $s:$ sterilization rate through air filters or air cleaners.

### Risk of infection in an indoor space through long-range transmission of airborne, aerosolized SARS-CoV-2 particles

For an average susceptible individual sitting in the restaurant, we calculated the risk of SARS-CoV-2 infection based on the number of viral PFUs that the individual is exposed to for the duration of a lunch event (1 h) or a dinner event (1.5 h). A susceptible individual is defined as a person who is disease-free at the start of lunch or dinner service and is at risk of contracting the disease while sitting in the restaurant. We assumed that if $N$ people are sitting in the restaurant for an event, based on the prevalence of disease in the surrounding community (denoted by $Pr$), there would be, on average, $N\cdot Pr$ infected individuals and $N-N\cdot Pr$ susceptible individuals for that event. We calculated the number of PFU units that a susceptible individual is exposed to during an event (denoted by ${nPFU}_{exposed}$) as follows:

$$nPFU_{exposed} = \int_{{t_{1} }}^{{t_{2} }} {N \cdot Pr \cdot C_{PFU} (t) \cdot Inhalation\_rate \cdot dt,}$$

where, ${t}_{1}$ and ${t}_{2}:$ represents respectively the starting and ending time of the event; $N\cdot Pr:$ represent the average number of infected people in the event; ${C}_{PFU}\left(t\right):$ represents the temporal concentration of viral PFU units (see the section “Temporal evolution of concentration of viable viral copies”); and $Inhalation\_rate$: Inhalation rate of 0.521 L/second for an average person with a sedentary activity person (e.g., sitting and speaking) [26].

We calculated the risk of infection (denoted by ${p}_{inf}$) for an average individual based on the number of viral PFUs the individual is exposed to during an event as follows:

$$p_{inf} = 1 - (1 - p_{d} )^{{nPFU_{exposed} }} ,$$

where ${p}_{d}$ represents the probability of infection per exposure to one viral PFU. We calculated ${p}_{d}$ as 0.0024 [95% Confidence Interval (CI) 0.0013–0.0053] based on an infectious dose 50 (ID50) of 280 (95% CI 130–530) PFUs [18, 27]. The ID50 indicates the number of viral particles required to cause infection in 50% of the individuals exposed to these particles. We varied the probability of infection of exposure to one viral PFU to test the cost-effectiveness for a range of airborne transmissibility values of SARS CoV-2 for past and potential future variants.

The above modeling approach considers only infection through the inhalation of accumulated aerosols, often referred to as the “long-range” mode of airborne transmission. Thus, transmission through the close-range mode—that is, when infectious aerosols were inhaled directly from the exhaled breath of an infected individual by a susceptible person in its vicinity—was assumed to be on par between the comparison arms and was disregarded.

### Costs

We modeled the cost of installing standalone air filtration units with HEPA filters, which trap ultrafine particles down to the sub-micrometer size [11]. Based on the restaurant’s size and cubic feet per minute (CFM) airflow of the standalone units, we calculated the number of units required to produce the equivalent of 12 ACH in the room. These units were assumed to be uniformly installed in the room to create different points of air disturbance.

We modeled direct and indirect costs of hospitalizations due to Covid-19 [28, 29]. For indirect costs of hospitalizations, we assumed a 21-day absence from work spanning the time spent in the hospital time spent at home after hospital discharge. We assumed 8 h of work lost per day at a value of $25/hour [30]. We assumed a 14-day quarantine for symptomatic infections for lost productivity and leisure time of 8 h per day. Future values were discounted at 3% [14, 17]. All costs were adjusted to 2020 US dollars (Table 2).

**Table 2:** Model input parameters for evaluating the cost-effectiveness of improving ventilation in commercial spaces for the prevention of SARS-CoV-2

Parameter	Base case value	Probability distribution
Number of people sitting in the restaurant at once	30	- (Changed in the sensitivity analysis from 20 to 40)
Average age of the people sitting in the restaurant	45	- (Changed in the sensitivity analysis from 35 to 55)
Probabilities and rates		
Probability of infection for one PFU unit exposed [based on ID50 of 280 (95% CI 130–530) PFU units] [18, 27]	0.0024	Beta (15.9592, 6633.707)
Proportion of asymptomatic cases among all exposed people (excluding the ones initially asymptomatic but became symptomatic eventually) [42]	0.25	Beta (18.5, 55.5)
Probability of long Covid-19 among symptomatic cases [43]	0.133	Beta (86.567, 564.3127)
Infection hospitalization rate [40]	Age-dependent: 0.019 for the average age of 45 years old	Beta (98.081, 5064.077)
Infection mortality rate [31]	Age-dependent: 0.001 for the average age of 45 years old	Beta (99.899, 99,799.1)
Relative rate of symptomatic infection with Delta among the fully vaccinated (relative rate of 0.22 is equivalent of 78% reduction in symptomatic infection; the value represents the average effectiveness of the BNT162b2 and ChAdOx1 nCoV-19 vaccines against Delta variant) [44]	0.22	Beta (14.8808, 52.7592)
Direct costs (US dollars in 2020 USD)		
Improving room ventilation rate to 12 ACH (by installing 5 standalone air filtration units with HEPA filters trapping ultrafine particles down to the sub-micrometer size that are uniformly installed in the room and produce an equivalence of 12 ACH for a 1000 ft^2^ space (each unit produces an airflow of 347 CFM and costs $750) [45]	$3750	Gamma (100, 0.02667)
Covid-19 hospitalization [28, 29]	$23,489	Gamma (100, 0.00426)
Indirect costs (U.S. dollars in 2020 USD)		
Covid-19 infection without hospitalization for symptomatic cases (losses of productivity over 2 weeks of self-isolation)	$2800	Gamma (100, 0.036)
Covid-19 hospitalization (losses of productivity over 3 weeks)	$4200	Gamma (100, 0.024)
Premature mortality due to Covid-19 (calculating losses of annual average wage of $50,000/year beyond the age at death of 45 years old in the base case model until the age of 65 years; future values were discounted at 3%)	$793,874	Gamma (100, 0.000126)
Health-related quality of life		
Losses of QALYs associated with a Covid-19 symptomatic case [31]	0.008	Beta (99.192, 12,299.81)
Losses of QALYs associated with a long Covid-19 infection [31]	0.034	Beta (96.566, 2743.61)
Losses of QALYs associated with a Covid-19 hospitalization [31]	0.020	Beta (97.970, 4776.154)
Losses of QALYs associated with a Covid-19 death (calculated based on an average age of 45 years at death, life expectancy of 80 years, age-dependent QALYs of the US general population, and discounting future values at 3%) [32]	18.33	Normal (18.33, 1.83)

*iD50* infectious dose 50; *CI* confidence interval; *PFU* plaque forming unit; *ACH*: air changes per hour; *HEPA* high efficiency particulate air; *CFM* cubic feet per minute (to measure airflow); *QALY* quality adjusted life year

### Health-related quality of life

We modeled losses of QALYs associated with a Covid-19 symptomatic infection and Covid-19 hospitalization [31]. A QALY is a metric capturing both longevity and health-related quality of life (HRQL). A QALY can be conceptualized as a year of life lived in perfect health and is calculated as the product of the life years remaining and the HRQL score. We also modeled changes in QALYs for the proportion of infected individuals who suffer from long-haul Covid-19 symptoms [31]. Finally, we modeled losses of QALYs associated with a Covid-19 premature death [32]. We discounted future values at 3% [14, 17].

### Analysis

We compared two interventions: (1) no improvement in the baseline ventilation rate of 0.8 ACH (‘status quo’), and (2) improving the room ventilation rate to 12 ACH. Our mathematical model was probabilistic and was developed in a Monte Carlo simulation of 5000 iterations, with each iteration randomly drawing from probability distributions of the input parameters. Table 2 shows the model inputs along with their probability distribution.

We performed our analyses for different conditions defined by the mean year-round prevalence of actively infectious cases in the surrounding communities where the restaurant is located and the proportion of patrons that are vaccinated. For the base case model, we assumed a 2% mean year-round prevalence of actively infectious cases in the surrounding community and a 70% full-vaccination rate among customers sitting in the restaurant, defined as 2 doses of an FDA approved vaccine in the US. We modeled the random daily incidence rate from a normal distribution and summed the daily incidence rates over the past 12 days to obtain the daily prevalence of actively infectious cases. This assumes an average of 12 days of infectiousness for an exposed individual beginning 2 days prior to symptom onset (for symptomatic cases) plus 10 days following the initial symptom onset [33, 34].

For the best-case scenario (minimum number of infections), we assumed a year-round prevalence of actively infectious cases of 0.1% in the surrounding community and a 90% full-vaccination rate among customers sitting in the restaurant.

For the worst-case scenario (maximum number of infections), we assumed a year-round prevalence of actively infectious cases of 3% in the surrounding community and a 0% full-vaccination rate among customers sitting in the restaurant.

The time horizon of the model was one year and the analytic horizon was lifetime. The outcomes of the model were incremental direct and indirect costs, infections averted, QALYs gained, and ICER for improving the ventilation rate. We also conducted one-way sensitivity analyses over all core input parameters of the model to measure the robustness of model outcomes against changes in these parameters.

## Results

### Base-case scenario

(2% prevalence of disease in the surrounding community where the restaurant is located and 70% full-vaccination rate among of restaurant customers). Improving the room ventilation rate to 12 ACH was associated with 54 [95% credible interval (CrI): 29, 86] infections averted in the standardized restaurant over one year. This produced cost savings of $152,701 (95% CrI: $80,663, $249,501), and 1.35 (95% CrI: 0.72, 2.24) incremental QALYs gained. Table 3 shows the complete results.

**Table 3:** Model outcomes including infections averted, incremental costs, incremental QALYs, and incremental cost-effectiveness ratio for upgrading the room ventilation rate from 0.8 to 12 ACH

	Airborne infections averted Mean (95% credible interval)	Net cost ($) Mean (95% credible interval)	Cost saving ($) Mean (95% credible interval)	Losses of QALYs Mean (95% credible interval)	Incremental QALYs^a^ gained Mean (95% credible interval)	ICER^b^ (95% credible interval)
Base-case scenario (2% prevalence of disease in the surrounding community where the restaurant is located and when 70% of the customers are vaccinated)						
Room ventilation rate of 0.8 ACH		$185,579 ($100,099, $300,430)		1.6 (0.85, 2.66)		
Improve room ventilation rate to 12 ACH	54 (29, 86)	$32,877 ($19,394, $50,877)	$152,701 ($80,663, $249,501)	0.25 (0.13, 0.42)	1.35 (0.72, 2.24)	− $113,126/QALY (dominant, dominant)
Best-case scenario (0.1% prevalence of disease in the surrounding community where the restaurant is located and when 90% of the restaurant customers are vaccinated)						
Room ventilation rate of 0.8 ACH		$6824 ($3524, $11,356)		0.06 (0.03, 0.11)		
Improve room ventilation rate to 12 ACH	2 (1, 4)	$4821 ($3930, $5865)	$2003 (− $881, $5968)	0.01 (0.01, 0.02)	0.05 (0.03, 0.09)	− $38,104/QALY (dominant, $30,503/QALY)
Worst-case scenario (3% prevalence of disease in the surrounding community where the restaurant is located and when no customer is vaccinated)						
Room ventilation rate of 0.8 ACH^c^		$544,521 ($298,694, $875,492)		4.35 (2.34, 7.14)		
Improve room ventilation rate to 12 ACH	135 (76, 213)	$89,243 ($50,540, $141,203)	$455,277 ($247,879, $734,424)	0.68 (0.37, 1.12)	3.66 (1.98, 6.02)	− $124,294/QALY (dominant, dominant)

The model outcomes are calculated for the base-case scenario (mean year-round prevalence of 2% in the surrounding community where the restaurant is located and when 70% of the customers are vaccinated), best-case scenario (mean year-round prevalence of 0.1% in the surrounding community where the restaurant is located and when 90% of the restaurant customers are vaccinated), and worst-case scenario (mean year-round prevalence of 3% in the surrounding community where the restaurant is located and when no customer is vaccinated)

Negative ICERs in this table represent a cost-saving scenario, meaning the comparator intervention saves money and improves health

*ICER* incremental cost-effectiveness ratio; *QALY* quality adjusted life year; *ACH* air changes per hour

^a^Quality-adjusted life years, which is equal to the product of the number of years of life gained and the health-related quality of life score

^b^The incremental cost-effectiveness ratio (ICER) is equal to the incremental cost divided by the incremental QALYs gained

^c^Air exchanges per hour. In this iteration of the model 0.8 is used as the baseline

### The best-case scenario

(0.1% prevalence of disease in the surrounding community where the restaurant is located and 90% full-vaccination rate among restaurant customers). This scenario reflects conditions far better than were observed for the United States for the pandemic through October of 2021. It was associated with cost savings of $2,003 (95% CrI: − $881, $5968) and 0.05 (95% CrI: 0.03, 0.09) QALYs gained.

### The worst-case scenario

(3% prevalence of disease in the surrounding community where the restaurant is located and 0% full-vaccination rate among restaurant customers). In this scenario, improving the room ventilation rate to 12 ACH was associated with 135 (95% CrI: 76, 213) infections averted, $455,277 (95% CrI: $247,879, $734,424) savings in costs, and 3.66 (95% CrI: 1.98, 6.02) increases in QALYs gained.

**Fig. 1:**
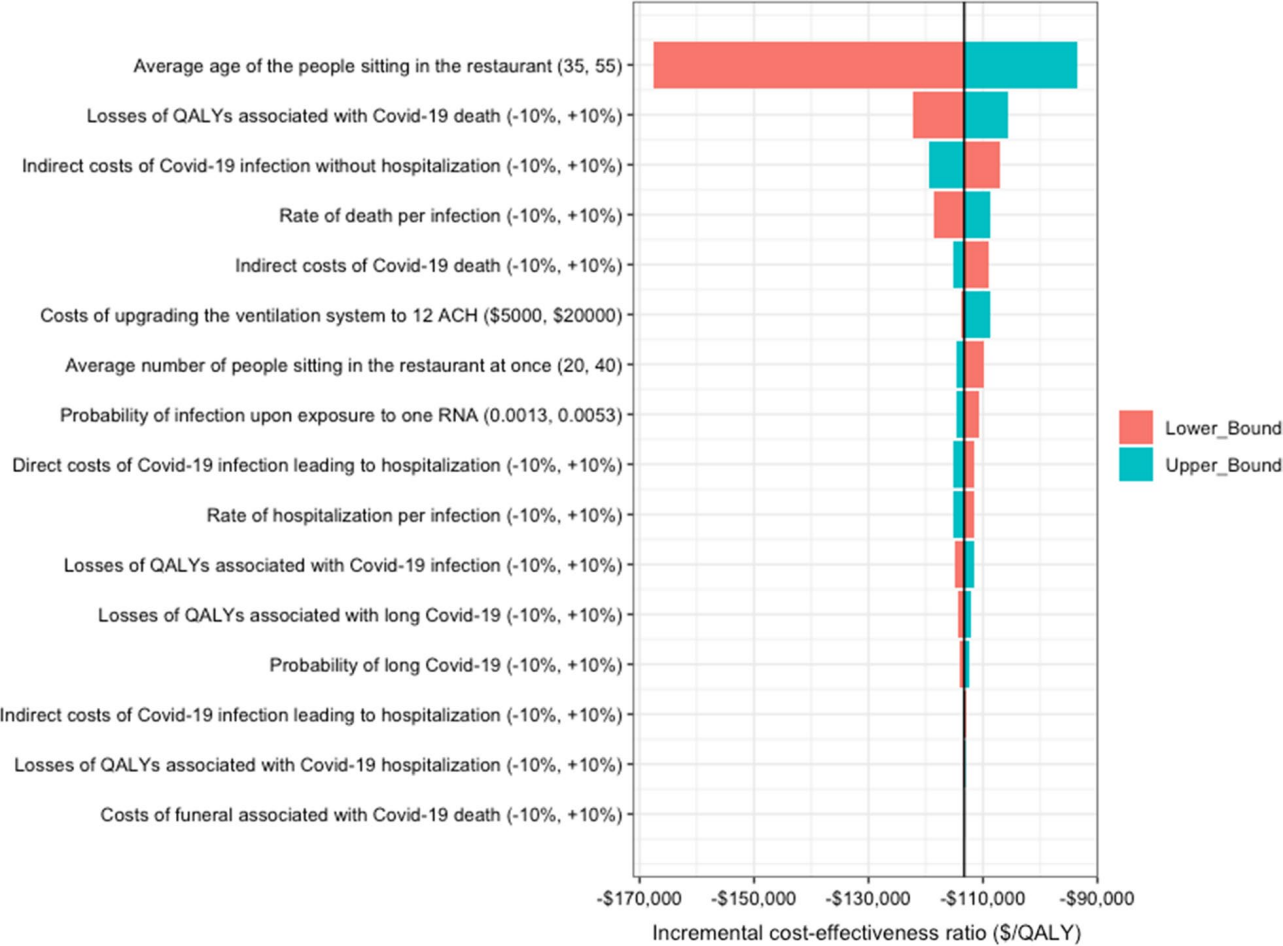
One-way sensitivity analysis (tornado diagram) for each of the core input parameters of the model. The range of each value represents the incremental cost-effectiveness ratio associated with varying model input parameters over a range of plausible values for the base-case model scenario (1000 ft^2^ restaurant space, 2% prevalence of actively infectious cases, a 70% vaccination rate, and an upgrade from 0.8 ACH to 12 ACH). *QALYs* quality-adjusted life years; *ACH* air changes per hour. Note in all the sensitivity analyses, improving the ventilation rate to 12 ACH saved money and improved health. Therefore, the negative incremental cost-effectiveness ratios on the x-axis can be interpreted as decreases in costs associated with improving the ventilation rate for one QALY gained. We encourage the reader to utilize the online model to obtain model outputs specific to the scenario that they wish to evaluate

Figure 1 shows the results of the one-way sensitivity analyses. In all sensitivity analyses, improving the room ventilation rate was cost-saving (dominant strategy) in the base-case scenario, meaning standalone air filtration units reduced airborne infections, increased QALYs gained, and resulted in savings for the commercial establishment.

**Fig. 2:**
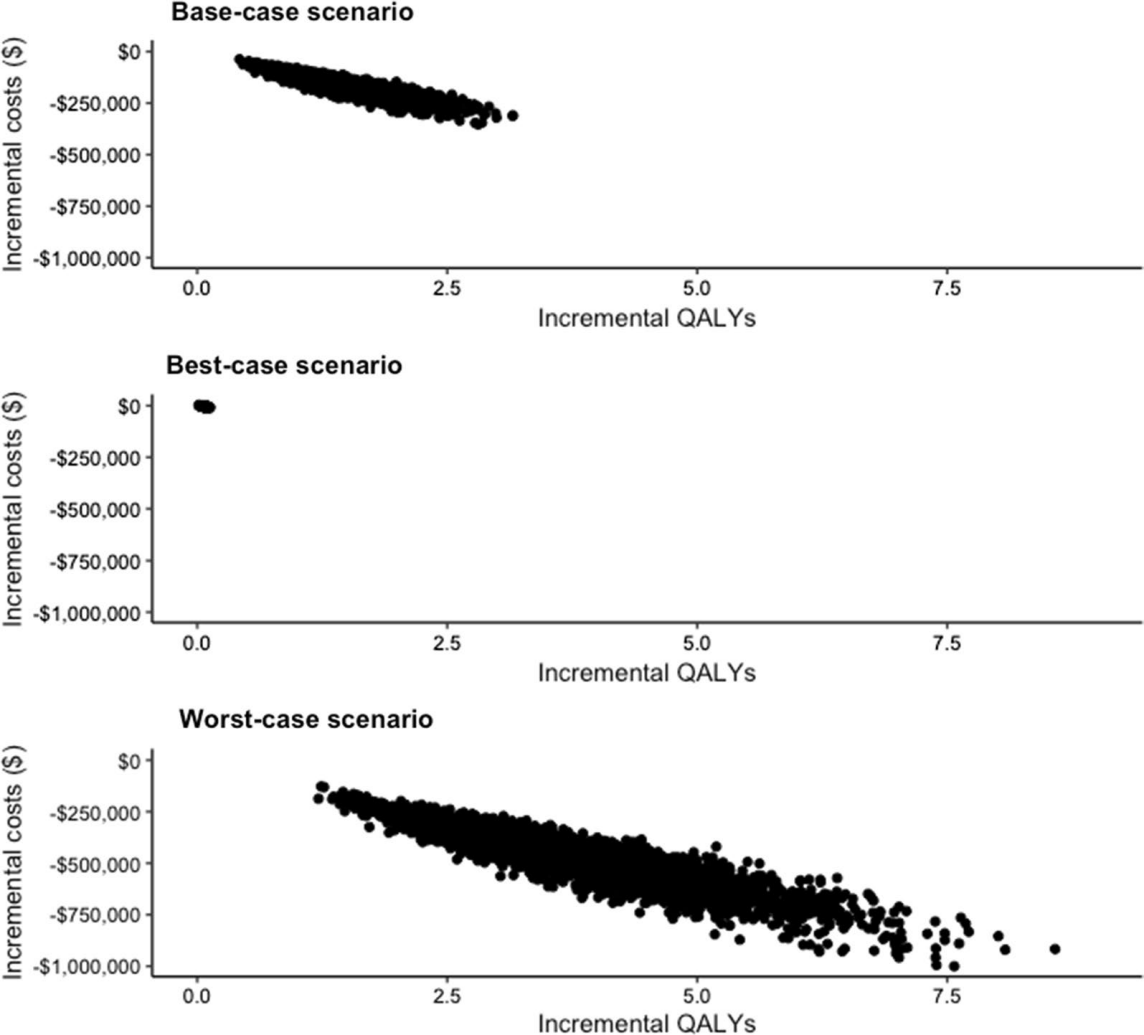
The cost-effectiveness plane representing the incremental costs versus incremental QALYs for improving the ventilation rate of an exemplary 1000 ft^2^ restaurant space to 12 ACH for: **A** the base-case scenario (mean year-round prevalence of 2% in the surrounding community where the restaurant is located and when 70% of the customers are vaccinated); **B** the best-case scenario (mean year-round prevalence of 0.1% in the surrounding community where the restaurant is located and when 90% of the restaurant customers are vaccinated); and (C) the worst-case scenario (mean year-round prevalence of 3% in the surrounding community where the restaurant is located and when no customer is vaccinated). The dots in the plot show the probabilistic runs of the Monte Carlo simulation with 5000 iterations. *QALYs* quality-adjusted life years; *ACH* air changes per hour

Figure 2 shows the probabilistic distribution of differential costs and differential QALYs of improving the room ventilation rate across all the Monte Carlo simulation runs. For the base-case and worst-case scenarios, in all the simulation runs, improving the ventilation rate resulted in cost savings and QALYs gained. Under the best-case scenario, 89% of the simulations resulted in cost savings and QALYs gained, and the 95% credible interval for ICER ranged from cost-saving (for intervention) to $30,503/QALY (< $50,000/QALY gained).

### Scenario analysis for a range of airborne transmissibility values

Because the variants of SARS CoV-2 are constantly changing (e.g., transition from Delta to Omicron BA.2), we conducted a scenario analysis to test the cost-effectiveness for different values of the airborne transmissibility of SARS CoV-2 (by changing the probability of infection per exposure to one viral PFU). When the airborne transmissibility was reduced by 50% (for variants with 50% lower rates of airborne spread), the standalone units were associated with $74,327 savings in costs and 0.67 increases in QALYs gained. When the airborne transmissibility was increased by 50% (for variants with 50% higher airborne spread), the standalone units were associated with $230,557 savings in costs and 2.02 increases in QALYs gained. Our online model allows the user to change the airborne transmissibility of SARS Cov-2 as new evidence emerges.

## Discussion

In this study, we evaluated the cost-effectiveness of improving ventilation in commercial indoor spaces using standalone HEPA filtration units as a method of preventing the transmission of airborne SARS-CoV-2. We built our probabilistic model using a Monte Carlo simulation so that the average model outcomes account for uncertainties and represent different ranges of variability in model input parameters and assumptions. Our probabilistic analyses showed that under all scenarios—even when the mean year-round prevalence of actively infectious cases was as low as 0.1% and 90% of the restaurant’s patrons were fully vaccinated—improving the ventilation rate of the indoor spaces by standalone air-filtration units would result in cost savings and QALYs gained. Our model was robust to changes across a range of inputs and assumptions, suggesting that policy mandates for HEPA filtration system use would be prudent in most situations.

There is a growing body of research modeling airborne transmission of SARS CoV-2 via aerosolized particles in indoor spaces [18, 35–39]. A recent study suggested that inhalation of aerosolized SARS-COV-2 particles is deemed to be an important source of transmission of the virus among the general population [36] and provided a theoretical model to quantify a safety cap for the number of occupants and the amount of time they should spend in an indoor space to reduce the airborne transmission of the virus. Other studies support the transmission of Covid-19 by aerosols [37], and suggest that indoor ventilation can significantly reduce infection [39]. However, the cost-effectiveness of such airborne preventive measures in a poorly ventilated indoor space is not clear, and hinges upon multiple factors. These include the community prevalence of disease, number of occupants and their time spent in the room, size of the room, and the proportion of occupants that are fully vaccinated. Our model allows for adjustable model input parameters that can be customized by the user using the accompanying online application (https://openupuniversities.shinyapps.io/Airborne_Transmission_Covid19/).

The underpinning risk transmission model has been previously vetted [18]. However, the parameter representing the airborne transmissibility, or the probability of infection for exposure to one viral PFU, must be modified according to emerging data from the current, predominant strain of SARS-CoV-2 [24]. Moreover, room geometry, temperature differences, or the motion of occupants can produce large changes in concentrations of aerosolized virus [20, 21].

Our study was limited in several ways. First, we modeled the hospitalization rate and mortality rate only as a function of age in line with the previous studies [31, 40]. In theory, however, hospitalization and mortality rates are also functions of other patient characteristics, such as gender, race, comorbidity, and socioeconomic status. Because the intent of our analysis was improving ventilation, we only modeled infections through inhalation of airborne, aerosolized viruses that may accumulate indoors, and we did not model infections via other pathways such as fomite transmission or inhalation of small and large droplets shortly after they are exhaled. This would result in an underestimate of cost savings and QALYs gained. In addition, our model does not provide data across different variants of SARS-CoV-2. Users of our online model must input transmission rates of the currently circulating strain to estimate the impact of standalone HEPA filtration units. We also note that, irrespective of whether aerosol transmission, droplet transmission, or fomite transmission is dominant, our model outcomes are not impacted.

## Conclusions

Even in the absence of SARS-CoV-2, poor ventilation systems in commercial spaces pose significant public health risks [41]. As the Covid-19 turns into an endemic disease, and as new pathogens emerge, it is critically important to set standards for ventilation of commercial spaces, even when they are in older buildings. In doing so, regulators must walk a fine line between setting standards that are too stringent to be economically viable for small business owners and the safety of the patrons of those establishments. We found that these systems are affordable from a governmental regulatory standpoint for most businesses. Moreover, as competition increases for novel air filtration systems, these systems are likely to fall in cost and increase in efficacy.

## Data Availability

We build an accompanying online dashboard for the model. Other data will be available upon request from the authors.
